# Spectroscopy Transmittance by LED Calibration

**DOI:** 10.3390/s19132951

**Published:** 2019-07-04

**Authors:** Daniel Carreres-Prieto, Juan T. García, Fernando Cerdán-Cartagena, Juan Suardiaz-Muro

**Affiliations:** 1 Department of Mining and Civil Engineering, Technical University of Cartagena, 30202 Cartagena, Spain; 2 Department of Information and Communications Technologies, Technical University of Cartagena, 30202 Cartagena, Spain; 3 Department of Electronic Technology, Technical University of Cartagena, 30202 Cartagena, Spain

**Keywords:** LED spectrophotometer, LEDs, water pollutants

## Abstract

Local administrations demand real-time and continuous pollution monitoring in sewer networks. Spectroscopy is a non-destructive technique that can be used to continuously monitor quality in sewers. Covering a wide range of wavelengths can be useful for improving pollution characterization in wastewater. Cost-effective and in-sewer spectrophotometers would contribute to accomplishing discharge requirements. Nevertheless, most available spectrometers are based on incandescent lamps, which makes it unfeasible to place them in a sewerage network for real-time monitoring. This research work shows an innovative calibration procedure that allows (Light-Emitting Diode) LED technology to be used as a replacement for traditional incandescent lamps in the development of spectrophotometry equipment. This involves firstly obtaining transmittance values similar to those provided by incandescent lamps, without using any optical components. Secondly, this calibration process enables an increase in the range of wavelengths available (working range) through a better use of the LED’s spectral width, resulting in a significant reduction in the number of LEDs required. Thirdly, this method allows important reductions in costs, dimensions and consumptions to be achieved, making its implementation in a wide variety of environments possible.

## 1. Introduction

Continuous and on-line quality monitoring of wastewater and stormwater is nowadays a primal objective to comply with the Urban Wastewater Treatment Directive (UWWTD 91/271/EEC), Bathing Water Directive (BWD, 2006/7/EC) and the Environmental Quality Standards Directive (EQS, 2008/105/EC). Variable wavelength spectroscopy is a non-destructive technique without the addition of chemicals reagents to wastewater that can be used for the quality monitoring and real-time control of sewers [1,2].

Several authors have proposed partial least squares calibration models for the indirect monitoring of chemical oxygen demand (COD) or total suspended solids (TSS) through Ultraviolet-Visible (UV–VIS) and Visible (VIS) spectroscopy for wastewater and stormwater combined sewer overflows (CSOs) [3,4,5]. Continuous and consistent data collection in sewers are presented. Spectrometer calibration experiments for total-COD in Reference [6] resulted in an absolute error in the range of 30–300 mg COD/L and a corresponding relative error in the range of 8–60%, where validation experiences led to underestimating high concentrations during daily peaks and in overestimating low concentrations during night periods [6,7].

Dissolved organic matter (DOM) was described by ratios of UV absorbance at 254 nm and 365 nm [8]. Different researchers have studied the absorbance at different wavelengths and its relation between different wavelength-absorbance as an indicator of the COD [8,9,10]. UV–Vis and VIS spectroscopy was performed in the wavelength range of 200–400 nm, where it was found that the ratio of absorbance at 250 and 365 nm was a good measurement for the COD correlation [10]. Ultraviolet-visible absorption spectra in two distinct spectral slope regions (275–295 nm and 350–400 nm) within log-transformed absorption spectra were used to compare DOM concentrations from contrasting water types [11].

Pollutants track-down during CSOs through cost-effective methods is demanded by municipalities to comply with legal requirements [12,13]. In the same way, industrial wastewater discharge control throughout the sewer systems is of great interest for administrations nowadays. Therefore, a large number of sensors are required for the real-time control of the CSOs and industrial discharges in the sewer systems during rainfall events and dry periods. To achieve this real-time monitoring of pollution in sewers, a robust, portable and cost-effective device is needed to continuously monitor quality in sewers.

LED lights have been selected as a replacement for traditional incandescent lamps, due to their low consumption, cost and the fact that they reach their operating regime almost immediately, which reduces downtime. The specific objective of this research is the development of a precision spectrophotometer, based on LED technology, and which is cost-effective, portable and with consumptions much lower than those provided by incandescent lamps [14], to enable them to be located in sewers systems massively. For the validation and acceptance of results obtained with LEDs, the first steps of the present work were dedicated to reproducing and comparing them to commercial equipment. The advantage of reproducing the footprint of a commercial spectrometer is the validation of the transmittance values with LED technology according to the configuration proposed in the present work, which avoids the use of an optical lens covering a wide range of wavelengths.

Therefore, the present research includes a calibration process that can be useful for the design of an innovative LED spectrophotometer capable of operating at a wide range of wavelengths. This availability of a greater working range (far beyond that of a turbidimeter that operates under a single wavelength, typically 860 nm) is expected to enable a more exhaustive analysis of a sample’s properties. Depending on the composition and concentration of wastewater and stormwater, variations in transmittance and absorbance values will be detected in certain regions of the working spectrum, which could enable a greater number of indirectly contaminating parameters to be determined.

Different types of LED based on light sources could be used in a spectrophotometer [15], but in practice not all are adequate for this purpose. In general, we can divide the types of LEDs into three categories: White light LED, RGB LED, and limited-bandwidth LED (Common LED).

In spectrophotometry, the use of white light for generating the working spectrum is a common practice. White light requires the use of optical elements, such as monochromators [16]. This supposes an increase in the cost of the equipment and in its dimensions, while making them more sensitive to mismatch as a result of impacts or vibrations, and thus not being suitable for all types of environments. There are studies [17] that raise the feasibility of using LED technology for the generation of white light, however, it must be pointed out that this type of LED semiconductor has a different emission spectrum than monochromatic white light. These are based on blue LEDs that are coated with a layer of phosphorus, which react with short wavelengths (blue), emitting low energy yellow light. Therefore, this type of LED is currently not the best solution for the generation of white light for spectrophotometry operations.

RGB LEDs were used to generate different wavelengths in the visible spectrum and through the combination of the three primary colors (Red, Green and Blue) with different levels of emission intensity [18]. An RGB LED is composed of three individual LEDs, that is, a red, green and blue LED that emit at specific wavelengths. However, in order to clarify the explanations, we shall refer to this type of light source as “RGB LED”, as these three LEDs are inside the same housing.

The idea of generating all the wavelengths that make up the visible spectrum using an RGB LED was shown to be unfeasible. It is based on an incorrect understanding of the human eye to affirm that certain wavelengths are being emitted [19], when in fact the primary colors are being emitted separately at different intensity levels, giving rise to an optical effect known as “rendering of color” [20]; television monitors are based on this. Although empirical equations exist between the values of *λ* and RGB, such as the Fortran code [21,22], which is one of the most used color generation systems in LED screens, this “color matching” cannot be used for the development of spectrophotometric equipment by only emitting the wavelengths corresponding to red: 625 nm; green: 525 nm; and blue: 460 nm [19].

Common LEDs represent the best option for the development of portable and cost-effective equipment. LED diodes are designed to emit in a limited range of wavelengths simultaneously (spectral width), so they are suitable for describing specific regions of the visible spectrum. Herein, in the present work we will refer to this type of LED.

The contributions of this work as follows: First, an innovative calibration process of the transmittance values for these LEDs without the use of optical elements such as lenses and diffraction matrix; thus reducing costs, dimensions and complexity. Second, increasing the valid range of wavelengths of each LED, in addition to its own peak wavelength. This allows the number of LEDs to be significantly reduced.

The rest of the article is organized as follows:

In Section 2, all the materials and methods used in this research work are explained. The section details the samples analyzed, the type of sensor used, as well as the commercial equipment based on incandescent lamps used as a reference during the calibration process. Likewise, the assembly carried out during the experiment is described, in order to provide a detailed guide to reproduce the results.

Section 3 focuses on the study of the use of LEDs as a viable alternative to incandescent lamps, detailing the limitations and problems associated with them. The need for a specific calibration process is evident to match the results obtained with reference commercial equipment based on incandescent lamps. The results obtained after the calibration process are also presented.

Finally, Section 4 discusses the considerations reached at the end of the research work.

## 2. Materials and Methods

### 2.1. Analyzed Samples

**Figure 1:**
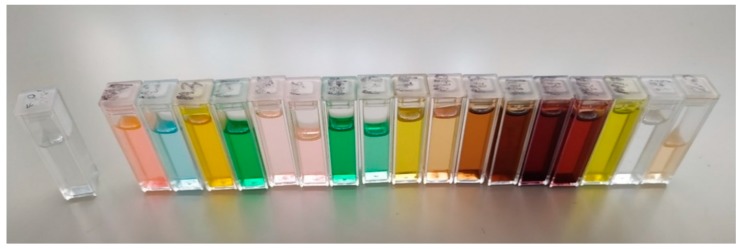
View of the 21 samples used in the present analysis.

In order to enable different values of transmittance to be compared, 21 different samples were prepared of different natures and concentrations, as shown in Figure 1. Moreover, Table 1 describes the different substances employed in each sample and their dissolution.

It should be noted that samples M11 and M12 were discarded from the present study due to the fact that all the incident light was absorbed, making the transmittance value null.

All these samples were stored in standard 12 × 12 × 50 mm plastic test tubes specially designed for spectrophotometry.

### 2.2. Sensors

Individual photodiodes were used in the present work rather than Charge-Coupled Device (CCD) sensors [23]. The individual photodiode is designed to capture a single beam of incident light [24,25]. These are sensitive to a wide range of wavelengths. This enables the dimensions and costs of the equipment to be reduced [26]. A single sensor usually collects a larger area in proportion to that of the photodiodes of the CCD.

**Figure 2:**
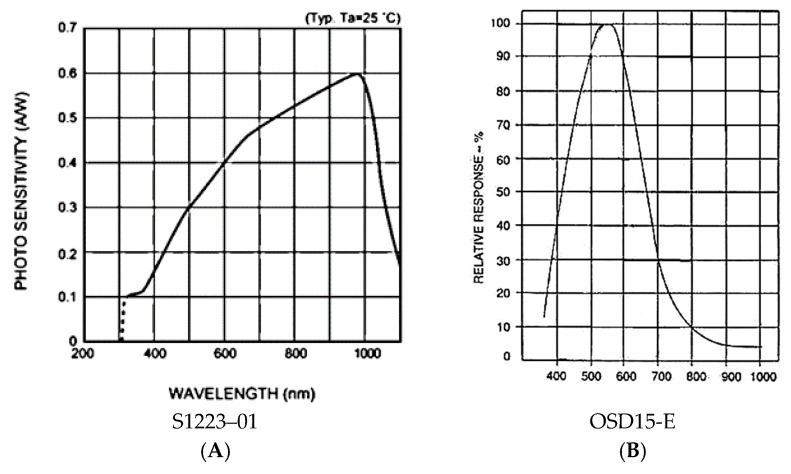
Sensitivity curves of the photodiodes (**A**) S1223–01 and (**B**) OSD15-E.

The individual photodiodes have a different spectral response depending on the incident wavelength. This is the sensor sensitivity. To study the effect of sensitivity in the measurements of transmittance, values at different wavelengths and two types of photodiodes with different sensitivities have been used to determine the most appropriate. These are S1223-01 [27] and OSD15-E [28], the spectral responses of which are shown in Figure 2.

### 2.3. Light Emitting Diode (LED)

**Figure 3:**
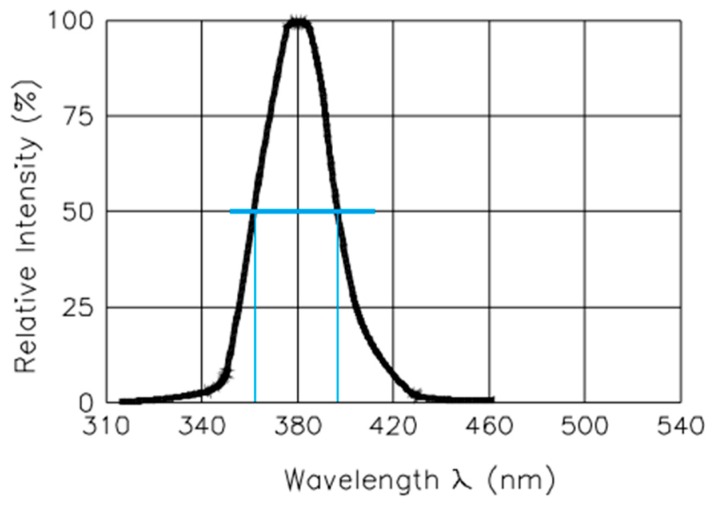
Emission spectrum of 385 nm LED (VAOL-5EUV8T4) [30].

As the source of light, traditional incandescent lamps were replaced by LEDs of 5 mm in diameter, which emit a fixed range of wavelengths determined by their manufacturing process. These are low energy consumers, cost-effective and are commercially available in a wide variety of peak wavelengths. This type of LED is designed to emit a narrow range of wavelengths simultaneously [29] and according to a normal distribution curve shape (Figure 3). The spectral width of an LED is defined by those wavelengths that have an emission intensity equal to or greater than 50%, as shown in Figure 3.

For instance, the VAOL-5EUV8T4 LED shown in Figure 3 [30], emits at 385 nm at its maximum intensity level (peak wavelength) and at 400 nm at the upper limit of the spectral width of the LED, also known as “center wavelength”, λ_0.5m_, which is defined as the wavelength halfway between the two points with a spectral density of 50% of the peak.

**Figure 4:**
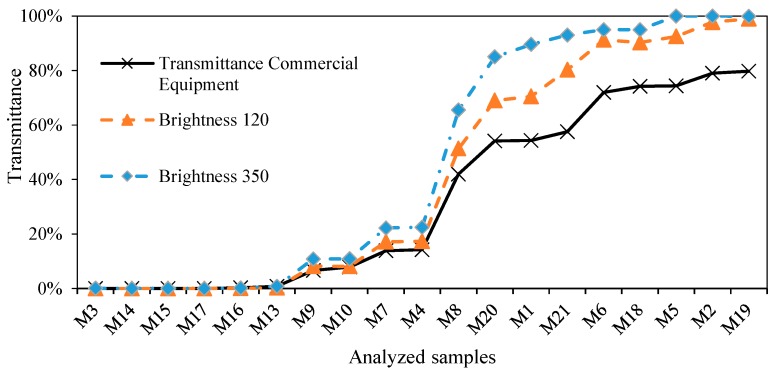
Transmittance comparison at 385 nm between commercial equipment and LED VAOL-5EUV8T4 [30] with two different brightnesses.

The whole range of wavelengths are emitted simultaneously. This results in higher values of transmittance than those provided by a spectrophotometer based on incandescent lamps. As shown in Figure 4 in the case of the 385 nm wavelength, the results provided by the LED reach deviations in the range of 14–20% with respect to the values obtained by means of the commercial equipment. Therefore, a calibration process to compensate for this deviation is required. Brightness selection will also be discussed in the present work.

To characterize the transmittance between 380 and 700 nm in a first attempt 59 LEDs were needed. At the end of this research work, through the optimization of the wavelength calibration and selection process we had reduced this number to 34 LEDs. These 34 LEDs are listed in the Appendix A (Table A1).

### 2.4. Hardware

In order to reduce the dimensions of the equipment and thus improve its portability, in the present research work it was decided to eliminate the use of any optical elements such as lenses, diffraction matrix or monochromators.

**Figure 5:**
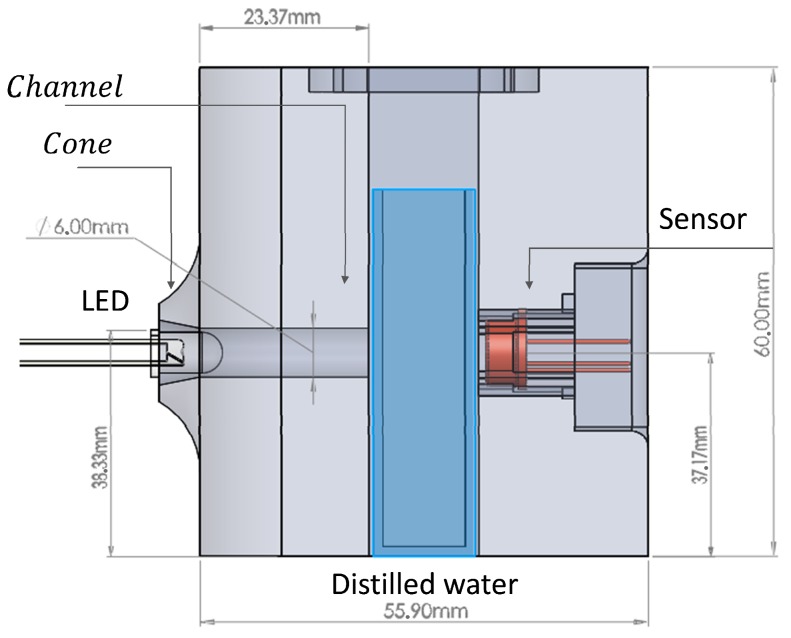
View of experiment assembly.

The proposed assembly is shown in Figure 5, constructed entirely in black thermoplastic Polylactic Acid (PLA) by a 3D printer. The tests carried out revealed that the most accurate results were obtained when the sensor (Figure 5 right) was as close as possible to the sample without touching it, and the light source (Figure 5 left) was at a distance of about 20 mm with reference to the test tube.

To minimize the dispersion of the LED light beam, it was necessary to have a cone-shaped entrance area to capture a greater amount of light, as can be seen in Figure 5. Additionally, a channel directing the beam to the center of the sample was needed. This channel needed to have a diameter slightly greater than the diameter of the LED so as not to obstruct the passage of light.

### 2.5. Reference Equipment

In the present work, all the results have been contrasted with the commercial equipment V-5000 VIS [31]. This has a working spectrum between 325 and 1000 nm, with a bandwidth of 4 nm, which complies with the following standards:

- ISO 22891: 2013: Determination of transmittance by diffuse reflectance measurement.
- ISO 10110-9: 2016: Preparation of drawings for optical elements and systems—Part 9: Surface treatment and coating.
- ISO 7887:2011: Water Quality—Examination and determination of color.
- ISO 9001 7.6 Control of monitoring and measuring equipment.

### 2.6. Brightness Level Definition

The definition of the brightness level of the LEDs is a key aspect of the entire calibration process. Higher values will cause sensor saturation, while low values may not allow the light to pass through the sample. To enable the brightness level to be varied, a driver [32] with a resolution of 16 bits has been used, capable of providing a maximum of 40 mA to each LED. The use of 16 bits provides a range of brightness levels between 0 and 4095 [33,34], the value at which the maximum level of illumination is reached (40 mA). Based on this, a brightness level of 100 corresponds to a current of 0.977 mA. Continuing with this example, the level of brightness 100 has been designated with the nomenclature T B100, used throughout this article. Low brightness levels are typically used when using high luminescence LEDs located a short distance from the sensor.

In this way, we consider that the amount of light detected (with a test tube of distilled water) corresponds to that emitted by the LED, ignoring the possible losses produced by the dispersion of the beam at the entrance of the channel (LED viewing angle).

### 2.7. Methodology

To carry out the calibration process that allows LED technology to produce results comparable to those obtained with incandescent lamps, it is necessary to carry out an adequate data collection. To do this, the transmittance values obtained by using LEDs of different peak wavelengths on different samples must be recorded (Table 1), so that they can be contrasted with the transmittance values obtained by means of the commercial equipment [31].

**Figure 6:**
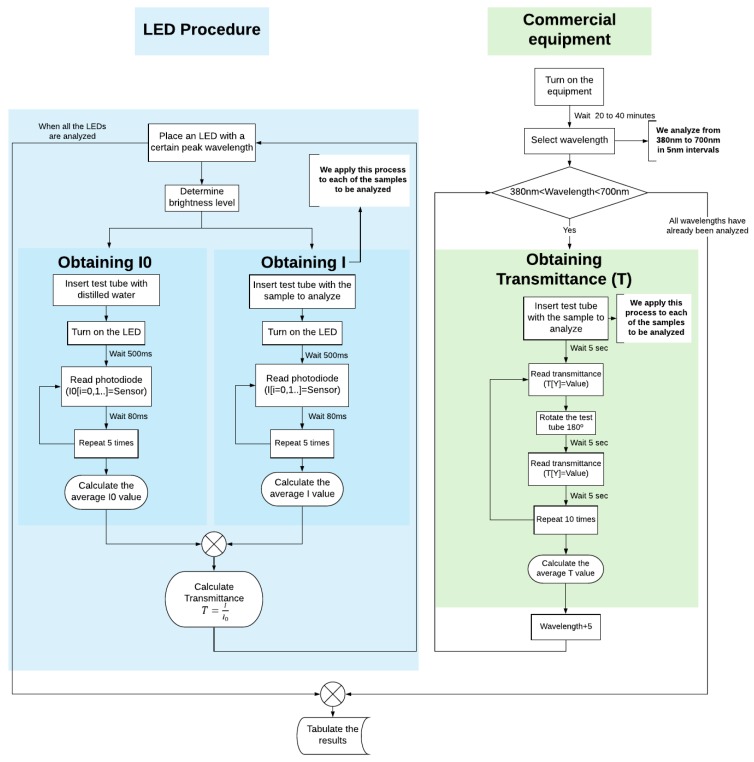
Scheme of the work methodology.

The methodology and procedure followed (with the help of software and electronic components) to obtain the data used for the measurement and calibration process are shown in Figure 6. This figure also shows the duration of the transmittance tests.

## 3. Result and Discussion

### 3.1. Preliminary Tests

**Figure 7:**
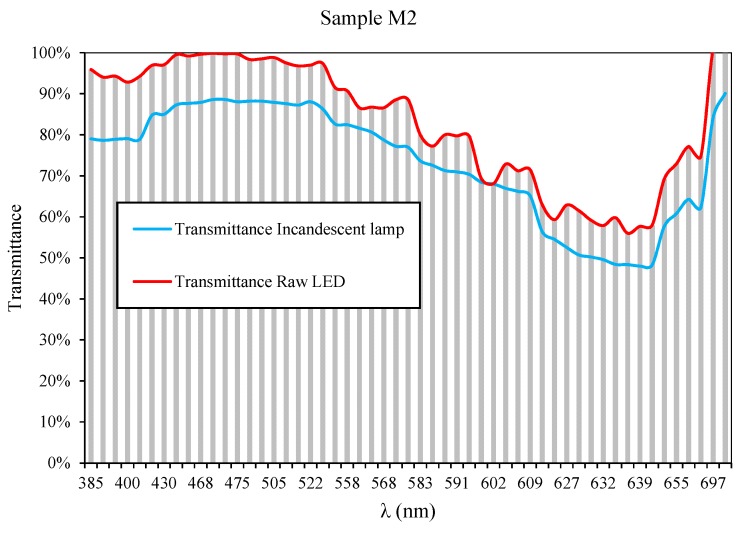
Comparison of the transmittance values obtained by a 5 mm LEDs spectral and those obtained by means of the commercial equipment.

As seen in Figure 7, when performing an analysis through 59 LEDs with $\lambda_{peak}$ between 385 and 700 nm, discrepancies in the transmittance values in comparison with the reference equipment based on incandescent lamps are observed. In this figure, each of the LEDs used has been denoted by a vertical line. Each LED operates at a wide range of wavelengths when it is traversing the sample (Figure 3), resulting in higher than expected transmittance values.

Moreover, the emission intensity of the LED plays a fundamental role in determining the transmittance values. Values which are too low could make the photons not pass correctly through a water sample with a certain turbidity. In contrast, too high a value would cause the saturation of the sensor, making the reading impossible. In conclusion, all these questions require the definition of a calibration process that compensates for this deviation in results.

### 3.2. LED Calibration

#### 3.2.1. Brightness Level Selection

When working with LEDs with different properties and from several manufacturers, it is not always possible to use a common brightness level for all of them. Each LED has different lighting capacities (Luminous Intensity) determined by its manufacturing process, in such a way that two different LEDs, powered by the same voltage and current can emit different brightness intensities. Therefore, if we change this current, we can also obtain different brightness levels.

**Figure 8:**
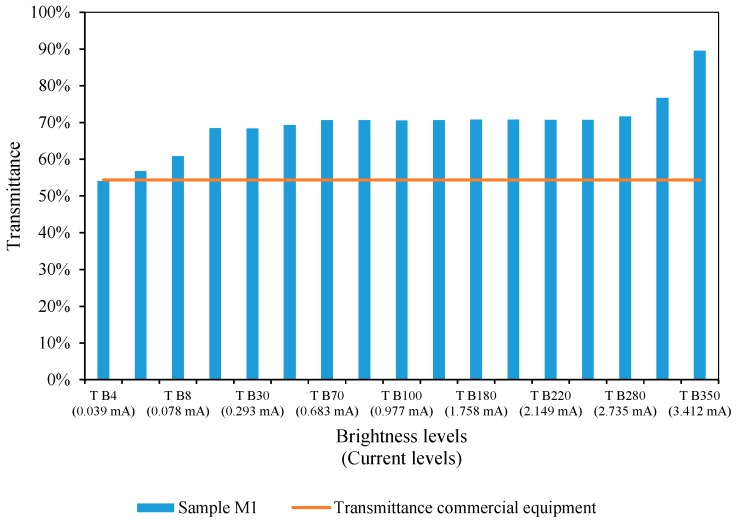
Effects of the brightness level in the transmittance measurement, using a 385 nm LED [30] on Sample M1.

If we take a known sample (M1), at different brightness levels with an LED of a certain peak wavelength (e.g., 385 nm), in Figure 8 we observe that the transmittance values remain constant between 68% and 70%, which corresponds to the brightness level obtained through a current from 0.073 mA to 2.051 mA. With high luminescence LEDs, at low currents, we achieve an enough brightness level to carry out the analysis of the samples. In this range of values, the transmittance values experiment little variation. As commented, the transmittance value is higher than the reference value of 54.35%, measured with the commercial equipment [31].

Each LED is able to emit at a maximum brightness level, determined by the manufacturer using the “Luminous Intensity” parameter, which is usually expressed in mcd (millicandelas) or lumens. In the case of the 385 nm LED used [30], its maximum luminous intensity is 100 mcd. Measuring the brightness level of the LED would require the use of expensive and complex equipment such as lux meters. Therefore, we have associated the brightness level with the current applied to the LED, which is a parameter that can be easily measured without having to use any additional equipment.

The current level selected must be within a certain range to achieve a stable transmittance value, even if it is greater than the expected one. As shown in Figure 3, the intensity at which the different wavelengths emit follows a normal distribution. The further away the wavelength is from the peak value, the lower its emission intensity. At low brightness values (low current), the effect of wavelengths is negligible. This can be seen in the transmittance value corresponding to brightness level 4 (T B4). We are using a 16-bit driver (a number between 0 and 4095) to control the current up 40 mA, so that, the brightness level designed as “T B4”, is obtained by applying a current of 0.039 mA $\left(\frac{4\;\times \;40\;\mathrm{mA}}{4095}=0.039\;\mathrm{mA}\right)$.

The transmittance value was 54%, fairly close to the expected one of 54.35%. However, although the results are close, such a low level of brightness is unsuitable for spectrophotometry operations. If the sample had a higher turbidity, photons could not pass through it. Therefore, the use of a current level within the stabilized values of transmittance is the best and most suitable option.

Although it is possible to select any current level lower than which causes the saturation of the sensor [35], it is highly recommended to select one which provides a response between 50–70% of the resolution of the sensor (with the distilled water sample). In our case, we are using a 16-bit resolution (or a number between 0 and 4095), that is, an *I*_0_ value between 511 and 716 (i.e., 2.5 and 3.5 V). This is the criterion followed here. In Figure 8 it would correspond to brightness levels between TB30 and TB280, i.e., 0.293 mA and 2.735 mA.

#### 3.2.2. Sensor Selection

We observe from Figure 2 that photodiode OSD15-E (Figure 2B) presents, for example, at 470 nm, a reference value $I_{0}$, (measured with a test-tube of distilled water), higher than S1223-01 (Figure 2A), since OSD15-E has a sensitivity greater than 50%, while S1223 barely manages to reach 25%.

However, the sensitivity of the sensor is not a critical parameter. If the reference value $\left(I_{0}\right)$ is greater, the light intensity crossing the sample to be analyzed (I), will increase proportionally. Therefore, the transmittance value $\left(T=\frac{I}{I_{0}}\right)$ will remain constant regardless of the type of sensor used. Based on the tests carried out, we can say that this statement is true provided that the sensor has a sensitivity of at least 5% for the wavelength to be analyzed. Finally, the selected photodiode was S1223-01, with a monetary cost three times lower than OSD15-E.

#### 3.2.3. Peak Value Calibration

**Figure 9:**
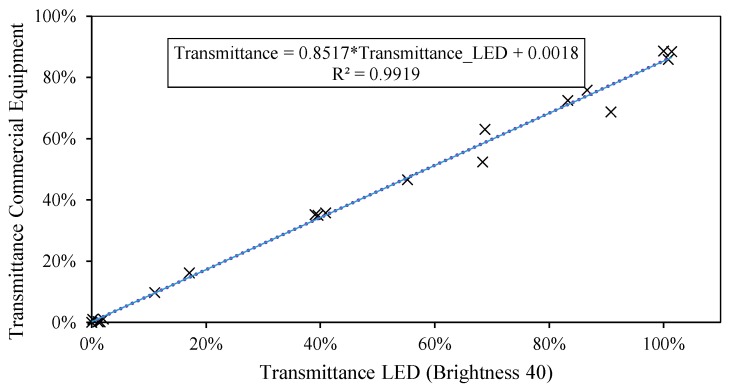
Scatter diagram between reference transmittance value and the most suitable brightness level (T40).

In preliminary tests, each LED was only used to obtain the transmittance for a unique value of wavelength, its peak wavelength. A linear relation is observed between the transmittance values registered with the LED and those corresponding to its $\lambda_{peak}$ from the reference equipment, as shown in Figure 9. The calibration of the peak value of the LED always produces a better fit, above 98%, as it is the highest wavelength intensity emitted by LED.

**Figure 10:**
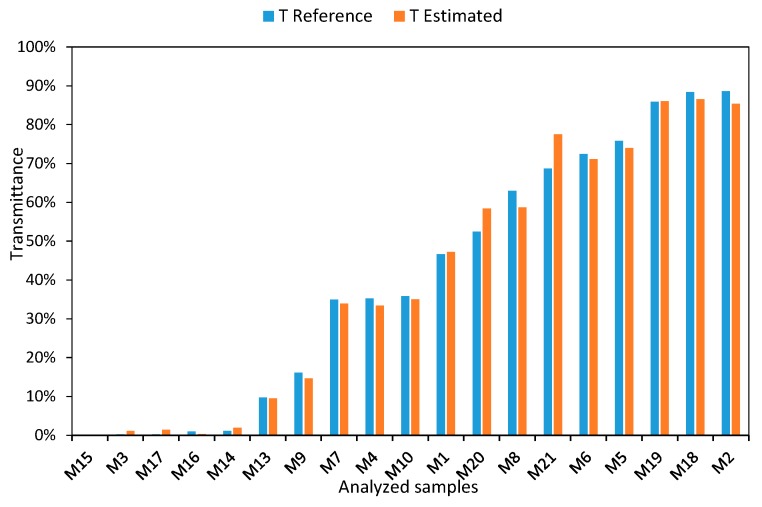
Comparison of calibration equations for 470 nm wavelength and brightness level 40.

After the fit, in Figure 10 we can observe good agreement between the reference values of transmittance obtained by the commercial equipment and those measured with a limited-bandwidth LED after the calibration process. To make it easier for the reader to follow, the samples have been ordered in an increasing way, according to the transmittance values obtained with the commercial equipment.

#### 3.2.4. Extension of the Working Range

In this section, we show how it is possible to use the spectral width of an LED to model the transmittance of other wavelengths in addition to that of the peak value.

**Figure 11:**
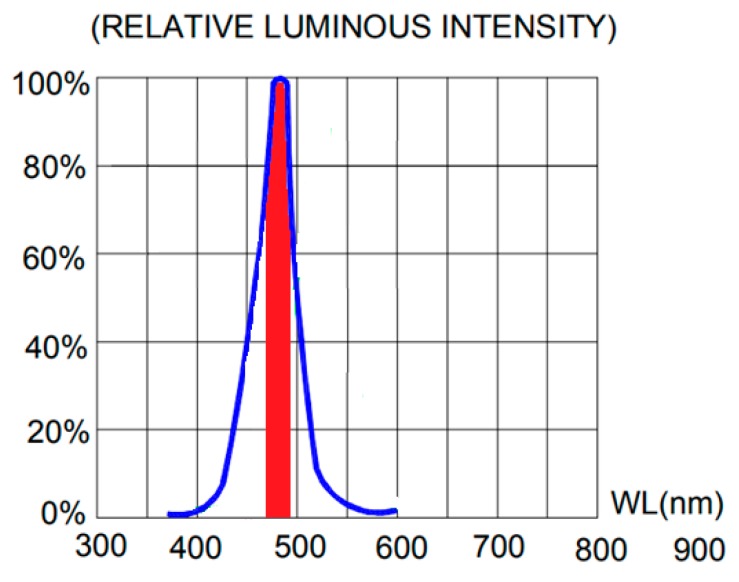
Representation of the useful working range of the 470 nm peak wavelength LED [36].

The tests carried out showed that the portion of the spectrum that can be used to extend the working range of a spectrophotometer is defined by those wavelengths between 10 and 20 nm over the peak value in the upper limit and the lower spectral width limit (LSWL). The LSWL is a parameter defined by the manufacturer (Table A1). These limits have been shown in Figure 11, using the LED with a 470 nm peak wavelength, as an example only.

As shown in Equation (1), despite being able to use the lower limit of the spectral width to calibrate the wavelengths, with a fit above 96%, from tests carried out we recommend working about 10 nm above this lower limit, in order to achieve a fit above 98%.

(1) $$\{\begin{array}{r} \lambda_{LSWL}<\lambda_{peak}<\;\lambda_{peak}+10\sim 20nm,\;\;0.96<R^{2}<0.99\\ \lambda_{LSWL}+10nm<\lambda_{peak}<\;\lambda_{peak}+10\sim 20nm,\;\;0.98<R^{2}<0.99 \end{array}$$

To illustrate this expression (1), we used an LED with a peak of 470 nm and a spectral width between 450 and 500 nm [36] to determine its spectrum working range. We start calibration at 450 nm wavelength, which represents the lower limit of the spectral width of the 470 nm LED, and represents the most unfavorable situation.

**Figure 12:**
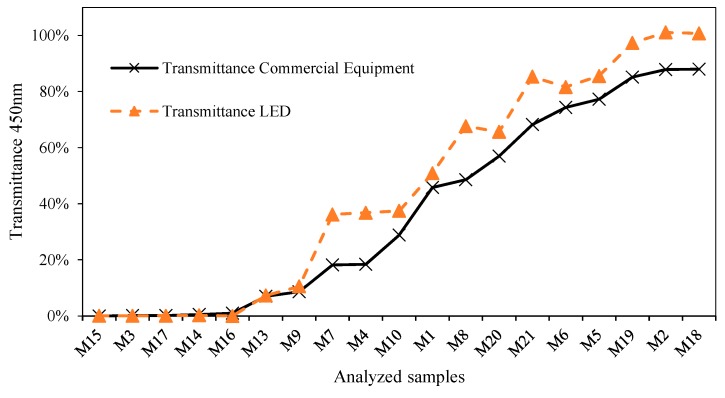
Comparison of transmittance obtained at 450 nm with the commercial equipment and the prototype developed based on an LED of dominant wavelength of 470 nm.

**Figure 13:**
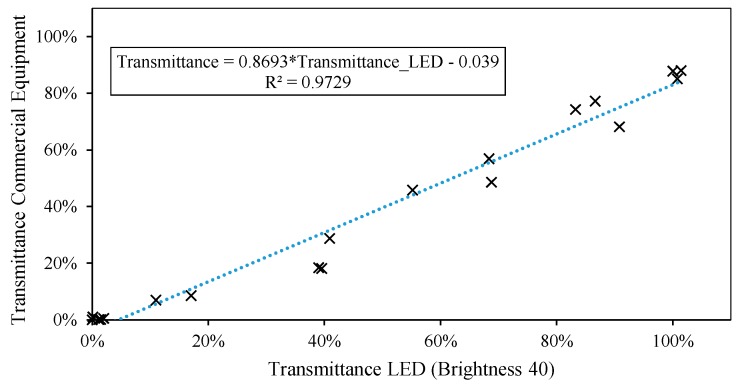
Scatter diagram between transmittance reference value at 450 nm and the most suitable brightness level (T40).

As seen in Figure 12, the trend is similar in both graphs [37]. Although some irregularities are presented in the scatter diagram of Figure 13, the goodness of the resulting fit is greater than 97%, showing that it is possible to use the portion of the spectrum included between the peak value of the LED and the lower limit of the spectral width. Extending this lower limit beyond that point will mean that the quality of the correlations will also be affected. If we take into account that the purpose of this calibration process is the development of spectrophotometric equipment with a comparable quality to the commercial one, a fit of lower than 97% is not desirable. Therefore, a wavelength placed 10 nm above the LWSL is recommended as being the lower transmittance capable that can be accurately reproduced by its corresponding $\lambda_{peak}$ LED.

**Figure 14:**
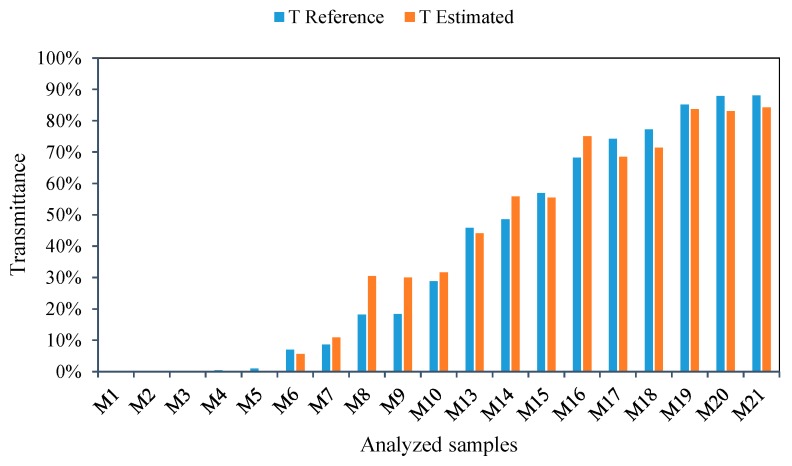
Comparison of calibration equations for 450 nm wavelength.

Figure 14 compares the transmittance values measured by LED after being correlated by the linear regression presented in Figure 13, and the transmittance values obtained with the reference equipment, where quite good agreement is observed. If we graphically represent the transmittance values calculated by this model, for very small values ($T_{B40}<0.0448)$, negative values are obtained, which makes no sense from the physical point of view. Therefore, this has been adopted as a criterion to consider any negative result as zero. This is adequate if we consider that the sample M14 had a reference transmittance value of 0.005. To make it easier for the reader to follow, the samples have been ordered in an increasing way, according to the transmittance values obtained with the commercial equipment.

It is also clear that wavelengths between this lower limit of the spectral width (450 nm) and the peak value can also be calibrated using the 470 nm LED, achieving an improvement in the goodness-of-fit as we approach the peak wavelength.

A certain *λ* can be calibrated through different LEDs, as long as it is included within its spectral width. Table 2 shows the case for the wavelength of 445 nm obtained with LEDs with different $\lambda_{peak}$.

As expected, the best fit is achieved for the 440 nm LED; however, the 438 nm LED provides a very similar fit.

**Figure 15:**
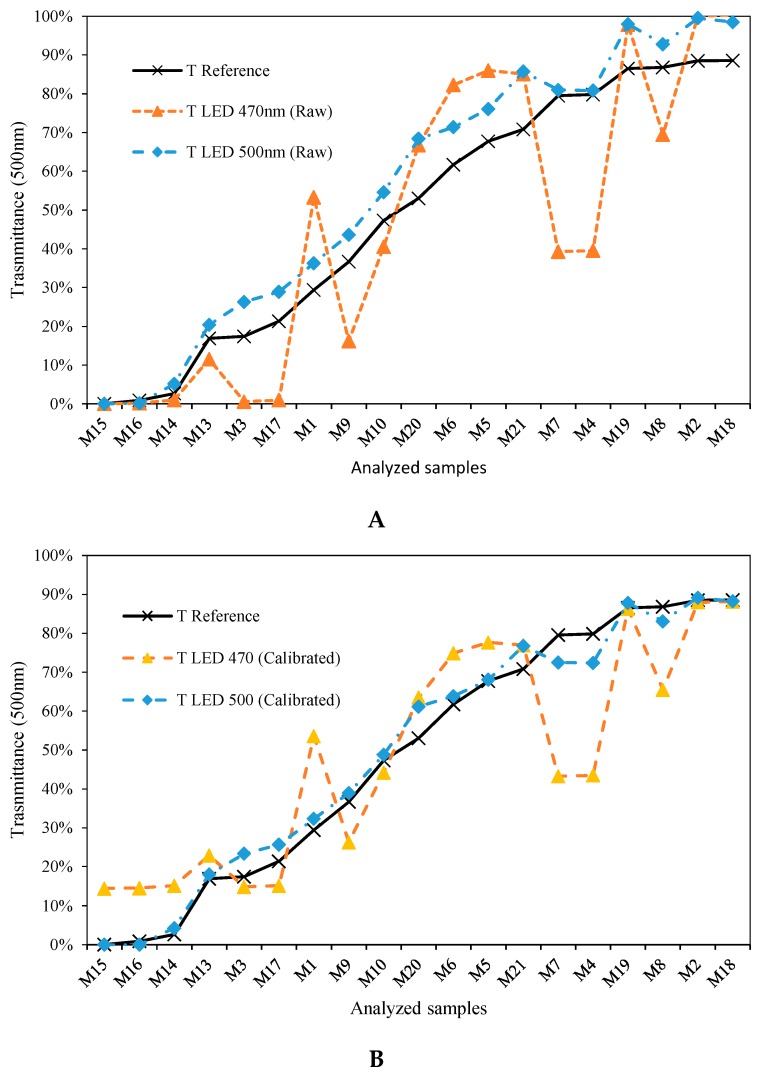
Comparison of the transmittance obtained at 500 nm with the commercial equipment and the prototype developed based on an LED with a peak wavelength of 470 nm and 500 nm. (**A**) Before the calibration process. (**B**) After the calibration process.

This raises the need to decide between calibrating a wavelength using an LED whose peak wavelength is the same (or very close) to that we seek to calibrate or using another LED that contains that wavelength in its spectral width. This is critical in order to be able to determine the effective working range of each diode. To illustrate this situation, we shall consider the calibration of an extreme case, the wavelength of 500 nm, which is the upper limit of the spectral width of the 470 nm LED [36]. The study was also carried out with LED SSL-LX5093UEGC [38], with a peak wavelength of 500 nm. The results are presented in Figure 15. This figure shows the comparison of the transmittance values obtained at 500 nm with LEDs of 470 nm and 500 nm of peak wavelength, before (Figure 15A) and after (Figure 15B) the calibration process. As can be seen, the results provided by the 500 nm LED are a closer fit to the reference values than those provided with the 470 nm LED. The first provides a fit of 98.97%, compared to 74.68% obtained with the 470 nm, which represents a worsening of 24%. This is because 500 nm is located at the upper limit of the spectral width of the 470 nm LED, i.e., 30 nm away, and is therefore expected to provide a worse response than an LED with a peak wavelength closer to 500 nm.

This result proves that we cannot use an LED whose peak wavelength is more than 30 nm from the one we seek to calibrate.

Nevertheless, as shown in Table 2, if the wavelength to be calibrated is at 20 nm or less with respect to the peak value of the LED (in the example from Table 2, 445 nm) it is possible to obtain a high goodness-of-fit. Proof of that is the LED with a peak wavelength of 428 nm, which can explain the 445 nm with an accuracy of 99%.

This shows that there is a limited capacity in terms of the wavelengths at which an LED can provide its spectral width. This process of verifying the useful spectral width of each LED has led us to the expressions shown in Equation (1).

#### 3.2.5. General Calibration Equation

The need to calculate the calibration lines for each of the wavelengths that we wish to add to our own equipment can result in a tedious and inefficient task. Any extension of the working spectrum would require the acquisition of new data and their comparison with the expected transmittance values.

Nevertheless, it is possible to find a generic expression that allows us to fit each of the desired wavelengths, independently of the LED used, as long as it includes that wavelength within its spectral width.

**Figure 16:**
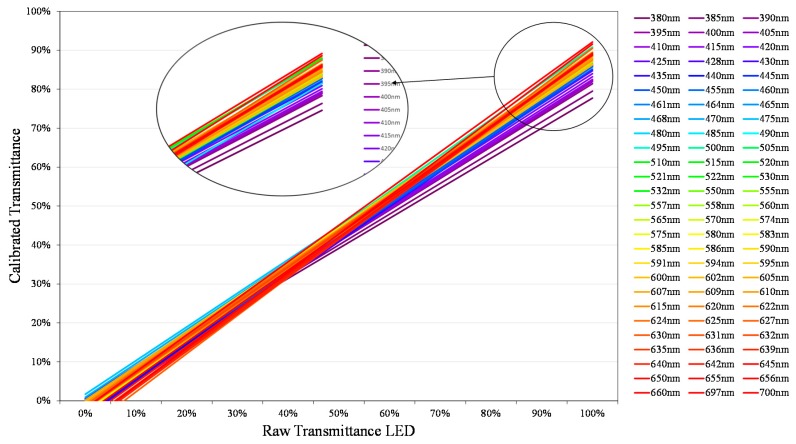
Calibration lines between 380 and 700 nm.

To illustrate the procedure followed to obtain generic calibration equations, all calibration lines calculated using the procedure described in the previous sections have been represented in Figure 16. In the present work, only wavelengths between 380 and 700 nm have been calibrated at 5 nm intervals, although it is possible to extend this range and reduce the bandwidth to 1 nm.

For a better understanding, each of the calibration lines has been represented with the color of its wavelength.

The calibration lines have a slope that changes depending on the wavelength. In the enlarged view of Figure 16, it can be observed that these are distributed in a very close way to how the visible spectrum does it [39]. However, the way the slope evolves changes depending on the wavelength range being analyzed.

**Figure 17:**
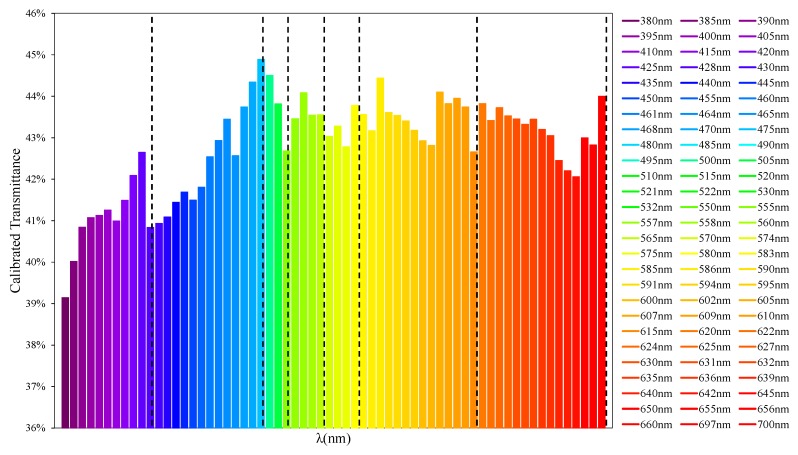
Transmittance values calculated at the tipping point of the calibration lines of Figure 16. Transmittance LED 50%.

To understand the behavior of the calibration lines (Figure 16), Figure 17 shows the transmittance values that would be obtained just at the tipping point of most graphs. In other words, the point where all the LEDs would provide a transmittance value (before calibration) of 50% for a certain sample. At this point, the behavior of the calibration lines of Figure 16 are most clearly observed.

**Figure 18:**
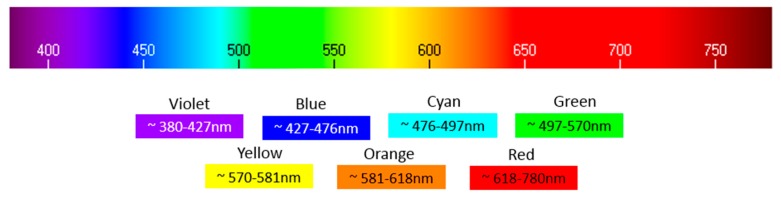
Visible light color spectrum.

As we can see in Figure 17, the graph forms groups. If we look at the wavelengths that delimit each group, we observe that these correspond to the different color groups that make up the visible light spectrum [40], the spectral limits of which are shown in Figure 18.

Based on these groups, we have determined the expressions that link the calibration line of Figure 16 to the wavelength. In the following we show only the procedure for determining the corresponding generic calibration equation for the “Blue” color group (427–476 nm), by means of an example.

Table 3 shows the calibration lines calculated for the wavelengths corresponding to the color blue, that is to say, between 427 and 480 nm, at intervals of 5 nm. When we work with a linear model (Figure 9, Figure 13 and Figure 16), the only parameters we need to estimate as a function of the wavelength are the slope *m* and the ordinate at the origin *n.*

**Figure 19:**
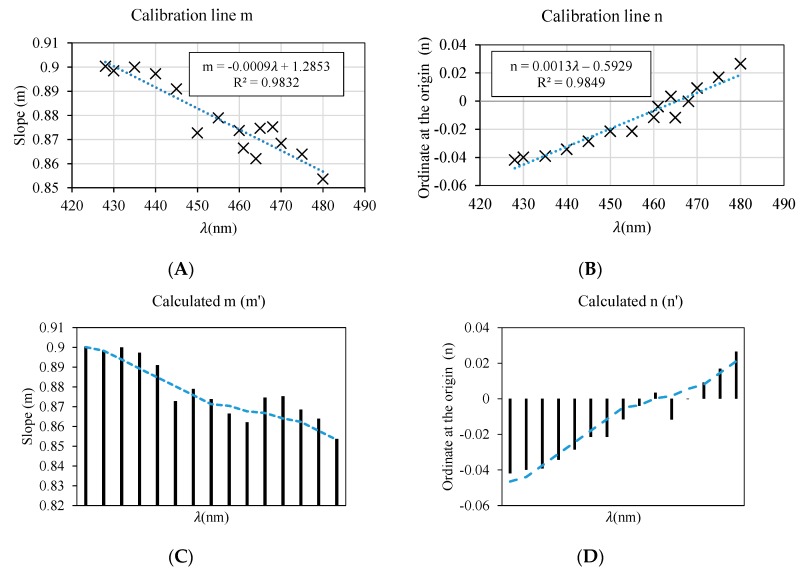
Calculation of the trend lines of the slope m and the ordinate at origin n according to λ. (**A**,**B**) are the scatter graphs used to enable the regression line (model) for the slope *m* and the ordinate at origin *n* to be calculated. (**C**,**D**) show the *m* and *n* values calculated through that model.

Through a scatter plot, it is possible to obtain the trend line that relates both parameters with respect to the value of *λ.* The results are shown in Figure 19.

Both the slope *m* and the ordinate at the origin *n* can be defined as a line whose slope depends on the wavelength we seek to calculate (Figure 19A,B). As shown in Figure 19C,D, the values of *m*′ and *n*′ calculated using this model are very close to the values obtained empirically using the manual calibration procedure, described in the previous sections.

From these data, it is possible to determine the expression that allows us to correlate the transmittance results obtained by the commercial equipment with respect to the values obtained by an LED, according to the wavelength.

In Equation (2), the generic calibration equations are shown for each of the color groups shown in Figure 18. It is important to emphasize that although the red color is up to 780 nm, we have only made the calibration up to 700 nm, since we do not have LEDs that emit at those wavelengths.

(2) $$\begin{array}{c} y_{380-427nm\;}=\;\left(0.0013\lambda +0.2941\right)*T\\ y_{427-480nm\;}=\;\left(-0.0009*\lambda +1.2853\right)*T+\left(0.0013*\lambda -0.5929\right)\\ y_{480-497nm\;}=\;\left(-0.00001*\lambda +0.9198\right)*T+\left(0.0043*\lambda -2.1638\right)\\ y_{497-570nm\;}=\;\left(-0.0004*\lambda +1.1273\right)*T+\left(0.0002*\lambda -0.1245\right)\\ y_{570-581nm\;}=\;\left(0.0578*\lambda -32.296\right)*T+\left(0.0221*\lambda +12.652\right)\\ y_{581-618nm\;}=\;\left(-0.018*\lambda +11.677\right)*T+\left(-0.0032*\lambda +1.8929\right)\\ y_{618-700nm\;}=\;\left(0.0006*\lambda +0.5462\right)*T+\left(-0.0001*\lambda +0.0277\right) \end{array}$$

### 3.3. Results

Using the calibration procedure described above, it is possible to achieve results very close to those obtained by using commercial equipment based on incandescent lamps.

This section shows the measured transmittance values for samples of different types and concentrations, along with the values provided by the commercial equipment.

**Figure 20:**
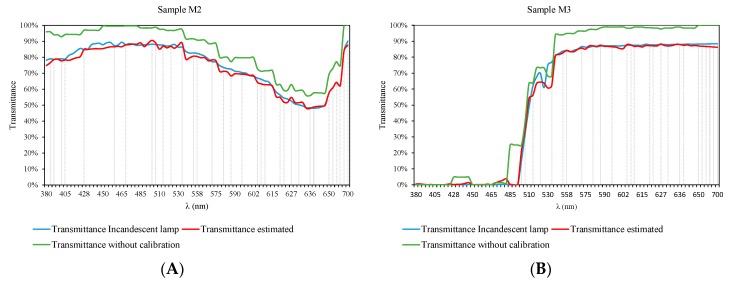

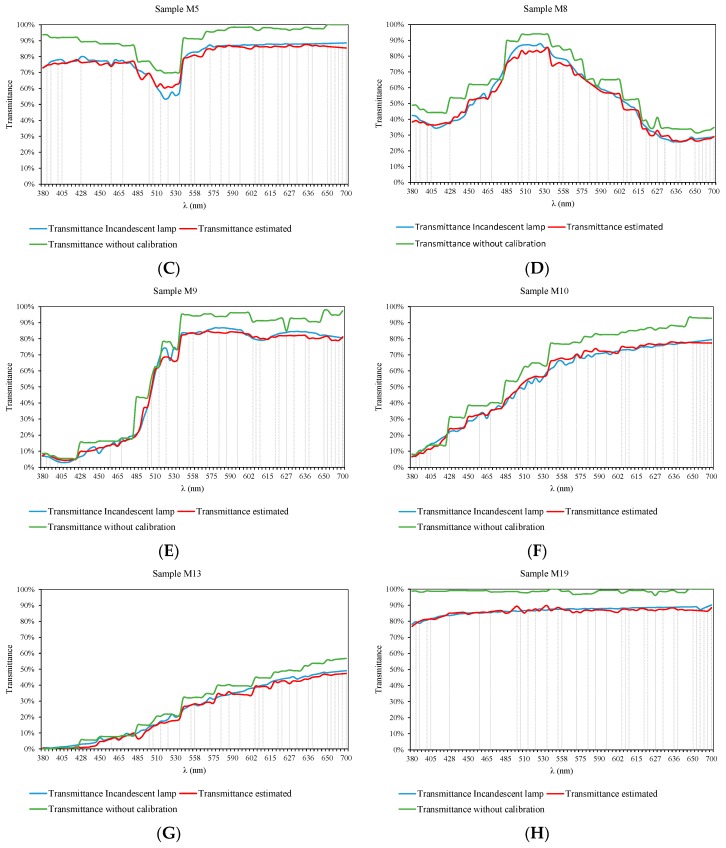
Comparative results of the transmittance values, before (green) and after (red) the calibration process for the following samples: (**A**) M2, (**B**) M3, (**C**) M5, (**D**) M8, (**E**) M9, (**F**) M10, (**G**) M13 and (**H**) M19.

As can be seen in Figure 20, there is good agreement between the two charts. Furthermore, each of the comparative graphs has been accompanied by the transmittance values obtained before the calibration process, in order to show the deviation in the results. This demonstrates that the calibration process described in the previous sections can produce results comparable to those obtained with incandescent lamps.

If we pay attention to Figure 20B–E, we observe that for wavelengths between 510 and 550 nm (which correspond to the green color spectrum) there are certain irregularities.

Such irregularity is due to the fact that after the calibration process, the fit was 96%, while the rest of the wavelengths obtained values above 98%.

This problem is probably due to the fact that the LEDs used to explain this region of the spectrum do not have the peak wavelength indicated by the manufacturer, since this problem has only been observed in the LEDs used for that wavelength range, whose peak wavelengths according to the manufacturer are 515, 531, 532, and 550 nm, respectively.

In each of the results graphs, the wavelength range explained by each of the 34 LEDs used after the calibration process has been delimited by vertical lines. Each vertical line presents an LED with a different peak wavelength.

**Figure 21:**
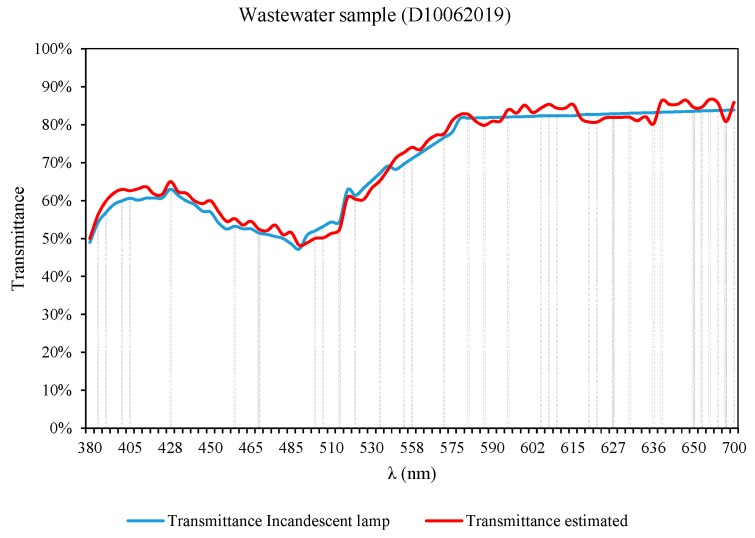
Wastewater sample analysis (D10062019).

The samples used to carry out the calibration process are representative of the transmittance values that can be presented by samples of wastewater from sewage networks. The wastewater samples can have transmittance values between 20–90% [41]. To show the adequacy of the calibration system, Figure 21 includes the results of the analysis performed with a sample of wastewater obtained from a treatment plant, which does not belong to the list of samples (Table 1) used during the calibration process.

In view of the results, it is possible to obtain a response comparable to that obtained by expensive commercial equipment based on incandescent lamps, through the use of LED technology, after a calibration process.

Several statistical indicators have been calculated to enable an analysis of how well the calibration process works. The quadratic mean error (RMSD) [42], and the error index, *Er*, are calculated according to Equations (3) and (4). These were calculated in the wavelength range of 380 to 700 nm, as seen previously in Figure 20.

(3) $$RMSD=\sqrt{\frac{1}{n}\sum_{i}^{n}{\left(T_{measured\_i}-T_{calculated\_i}\right)}^{2}}$$

(4) $$Er\left(\%\right)=\|\frac{\sum_{i}^{n}\left(T_{measured\_i}-T_{calculated\_i}\right)}{\sum_{i}^{n}T_{measured\_i}}*100\|$$

where *Er* is the error index (%); *n* is the number of wavelength (in our case, it takes the value of 81); $T_{measured}$ and $T_{calculated}$ are the values of transmittance obtained through the commercial equipment [31] and own design based on LED technology (Figure 5), respectively.

As we can see in Table 4, we obtained an RMSD very close to zero for all the samples. This shows that the transmittance values obtained through commercial equipment and those obtained through the calibration process described in this research work are very close to each other.

Proof of this lies in the error values, *Er*. For all the samples, the error has always been lower than 5%, and in almost all cases was less than 1%. That is an error index comparable to that the equipment based on incandescent lamps.

It should be noted that not all the LEDs used have taken full advantage of their useful spectral width. Although the range of the spectral width shown in Equation (1) ensures us a goodness-of-fit above 97%, it has been chosen not to take all the range of the diodes, using more LEDs in order to achieve a fit close to 99%. This has resulted in a total of 34 LEDs to calibrate 81 wavelengths.

## 4. Conclusions

In this paper, we have shown that it is possible to use LED technology as a replacement for traditional incandescent lamps, for the development of spectrophotometers. To achieve this, it is necessary to carry out a calibration process the compensate for the effect produced by the multiple wavelengths that the LEDs emits at the same time (spectral width).

Therefore, we can conclude that the calibration procedure described enables us to achieve results comparable to those obtained by incandescent lamps. Proof of that lies in the fact that the error obtained has been less than 5%, and was around 1% in most cases analyzed (Table 4).

The methodology proposed allows us to:

(i) Obtain results comparable to those provided by commercial equipment based on incandescent lamps, without using any type of optical elements such as lenses or diffraction matrix that would make the equipment more expensive;
(ii) Extend the working range of each LED, covering the whole visible spectrum using a smaller number of LEDs;
(iii) Reduce the dimensions, costs and sampling times of the equipment, which are vital aspects for the development of low-cost autonomous systems designed to measure in any type of environment.

This calibration procedure allows anyone to develop their own spectrophotometry equipment. This means that everyone can have access to this type of equipment at a small fraction of the cost of commercial equipment.

This procedure has enabled us to achieve a working range between 380 and 700 nm by using just 34 LEDs that cover different areas of the visible spectrum, with a resolution of 5 nm.

That opens the door to the development of new systems based on this technology, lowering the cost of equipment while reducing its size and consumption, enabling the creation of autonomous equipment that can run on batteries in any environment.

This type of systems can be very useful for the detection of unauthorized discharges, as the system can be placed in any environment, monitoring 24 h a day. It is therefore a tool against fraud and can contribute to environmental protection.

All this research work will allow us to have real-time control of water quality in sewer systems during rainfall events and dry weather periods, through a portable and cost-effective device to analyze the contaminant load present in wastewater.

## Figures and Tables

**Table 1:** Analyzed samples.

Designation	Substance	Dissolution
M0	Distilled water	100%
M1	Red food coloring 40	50%
M2	Blue food coloring	50%
M3	Yellow food coloring	40%
M4	Washing machine detergent	50%
M5	Red food coloring 40	75%
M6	Red food coloring 40	55%
M7	Washing machine detergent	65%
M8	Washing machine detergent	75%
M9	Kitchen oil	100%
M10	Vinegar	90%
M11	Milk	100%
M12	Milk	50%
M13	Soluble coffee	75%
M14	Soluble coffee	50%
M15	Red wine	100%
M16	Red wine	50%
M17	Blue and Yellow food coloring	20–80%
M18	Blue food coloring	30%
M19	Sea water	100%
M20	Tea	80%
M21	Fabric softener	40%

**Table 2:** 445 nm fit quality comparison using different LEDs.

λ	$\lambda_{peak}\;\mathrm{LED}$	$R^{2}$	Calibration Equation
445 nm	428 nm	0.9924	y = 0.891x − 0.0286
	430 nm	0.9927	y = 0.8868x − 0.0298
	435 nm	0.9939	y = 0.8862x − 0.0305
	438 nm	0.9971	y = 0.8853x − 0.0304
	440 nm	0.9978	y = 0.8852x − 0.0301
	458 nm	0.9803	y = 0.8847x − 0.0314
	461 nm	0.9714	y = 0.8832x − 0.0323

**Table 3:** Calibration straight lines between 427 and 480 nm (Blue).

λ	Calibration Equation	m	n	$R^{2}$
428	y = 0.9004x − 0.0418	0.9004	−0.0418	0.9892
430	y = 0.8985x − 0.0399	0.8985	−0.0399	0.9906
435	y = 0.9x − 0.0391	0.9	−0.0391	0.9911
440	y = 0.8973x − 0.0342	0.8973	−0.0342	0.9917
445	y = 0.891x − 0.0286	0.891	−0.0286	0.9924
450	y = 0.8728x − 0.0214	0.8728	−0.0214	0.9918
455	y = 0.879x − 0.0214	0.879	−0.0214	0.9941
460	y = 0.8738x − 0.0115	0.8738	−0.0115	0.9973
461	y = 0.8665x − 0.0039	0.8665	−0.0039	0.9986
464	y = 0.8621x + 0.0034	0.8621	0.0034	0.9981
465	y = 0.8747x − 0.0117	0.8747	−0.0117	0.9972
468	y = 0.8753x − 0.0002	0.8753	−0.0002	0.9978
470	y = 0.8685x + 0.0092	0.8685	0.0092	0.9953
475	y = 0.864x + 0.0169	0.864	0.0169	0.9875
480	y = 0.8537x + 0.0265	0.8537	0.0265	0.9628

**Table 4:** RMSD and Error index.

Sample	RMSD	*Er* (%)
M2	0.02062086	1.451423749
M3	0.028591905	0.962997523
M5	0.024032501	0.990803799
M8	0.027103038	2.135421037
M9	0.023634811	1.473856032
M10	0.019272554	1.426693178
M13	0.017883052	4.921374084
M19	0.013081104	0.986143228
D10062019	0.020993826	−1.152994953

## Appendix A

**Table A1:** LEDs used.

ID	$\lambda_{Peak}\;(\mathrm{nm})$	Spectral Width		Reference	Datasheet
		Lower Limit (nm)	Upper Limit (nm)		
1	385	360	400	VAOL-5EUV8T4	www.mouser.es
2	390	388	392	UV5TZ-390-15	www.mouser.es
3	395	393	398	UV5TZ-395-15	www.mouser.es
4	400	398	400	UV5TZ-400-15	www.mouser.es
5	405	395	415	VAOL-5EUV0T4	www.mouser.es
6	428	400	440	4304H6	www.mouser.es
7	461	440	480	151053BS04500	www.mouser.es
8	468	456	480	LTL2P3TBK5	www.mouser.es
9	500	475	520	SSL-LX5093UEGC	www.mouser.es
10	505	487	518	LTL2V3TCYK2	www.mouser.es
11	515	497	531	WP7113ZGCK	www.eu.mouser.com
12	521	505	536	HLMP-HM74-34CDD	www.broadcom.com
13	532	506	544	LTL2P3TGZ2KS	www.mouser.es
14	557	543	575	WP7113PGC	www.eu.mouser.com
15	558	500	570	HLMP-C615-G0001	www.broadcom.com
16	574	585	602	TLCYG5100	www.mouser.es
17	583	550	610	521-9466F	www.mouser.es
18	586	565	615	521-9271F	www.uy.mouser.com
19	594	587	600	HLMP-EL1A-Z1KDD	www.broadcom.com
20	605	590	620	SLI-580DT3F	www.mouser.es
21	607	581	623	WP7113NC	www.kingbrightusa.com
22	609	600	618	HLMP-EJ15-SV000	www.mouser.es
23	622	612	632	TLCR5200	www.mouser.es
24	624	597	648	C503B-RBN-CX0Y0AA1	www.mouser.es
25	627	600	650	WP57EYW	www.mouser.es
26	631	623	645	151053RS03000	www.mouser.es
27	636	625	648	SSL-LX5093SIC	www.eu.mouser.com
28	639	630	650	LTL2H3KRK	www.mouser.es
29	650	635	663	HLMP-4101	www.mouser.mx
30	655	650	675	WP7113SRC/DU	www.mouser.es
31	656	645	665	VAOL-5GAE4	www.eu.mouser.com
32	660	648	675	LTL-4268-H3	www.mouser.es
33	697	650	743	LTL-4213	www.digikey.com
34	700	655	730	SSL-LX5093HD-TR	www.lumex.com

## References

[1] Thomas O., Burgess C. (2017). UV-Visible Spectrophotometry of Water and Wastewater.

[2] Bourgeois W., Burgess J.E., Stuetz R.M. (2001). On-line monitoring of wastewater quality: A review. J. Chem. Technol. Biotechnol. Int. Res. Process Environ. Clean Technol..

[3] Chen B., Wu H., Li S.F.Y. (2014). Development of variable pathlength UV-Vis spectroscopy combined with partial-least-squares regression for wastewater chemical oxygen demand (COD) monitoring. Talanta.

[4] Brito R.S., Pinheiro H.M., Ferreira F., Matos J.S., Lourenço N.D. (2014). In situ UV-Vis spectroscopy to estimate COD and TSS in wastewater drainage systems. Urban Water J..

[5] Hochedlinger M., Kainz H., Rauch W. (2006). Assessment of CSO loads–based on UV/VIS-spectroscopy by means of different regression methods. Water Sci. Technol..

[6] Gruber G., Winkler S., Pressl A. (2004). Quantification of pollution loads from CSOs into surface water bodies by means of online techniques. Water Sci. Technol..

[7] Gruber G., Bertrand-Krajewski J.L., Beneditis J.D., Hochedlinger M., Lettl W. (2006). Practical aspects, experiences and strategies by using UV/VIS sensors for long-term sewer monitoring. Water Pract. Technol..

[8] Yuan D.H., He J.W., Li C.W., Guo X.J., Xiong Y., Yan C.L. (2019). Insights into the pollutant-removal performance and DOM characteristics of stormwater runoff during grassy-swales treatment. Environ. Technol..

[9] Weishaar J.L., Aiken G.R., Bergamaschi B.A., Fram M.S., Fujii R., Mopper K. (2003). Evaluation of specific ultraviolet absorbance as an indicator of the chemical composition and reactivity of dissolved organic carbon. Environ. Sci. Technol..

[10] He X., Xi B., Wei Z., Guo X., Li M., An D., Liu H. (2011). Spectroscopic characterization of water extractable organic matter during composting of municipal solid waste. Chemosphere.

[11] Helms J.R., Stubbins A., Ritchie J.D., Minor E.C., Kieber D.J., Mopper K. (2008). Absorption spectral slopes and slope ratios as indicators of molecular weight, source, and photobleaching of chromophoric dissolved organic matter. Limnol. Oceanogr..

[12] Irvine K., Rossi M.C., Vermette S., Bakert J., Kleinfelder K. (2011). Illicit discharge detection and elimination: Low cost options for source identification and trackdown in stormwater systems. Urban Water J..

[13] Panasiuk O., Hedström A., Marsalek J., Ashley R.M., Viklander M. (2015). Contamination of stormwater by wastewater: A review of detection methods. J. Environ. Manag..

[14] Jones K.P. (2016). New lamps for old—Recent progress in UV sources for absorption detectors in liquid. TRAC: Trends Anal. Chem..

[15] O’Toole M., Diamond D. (2008). Absorbance based light emitting diode optical sensors and sensing devices. Sensors.

[16] Visconti P., Primiceri P., de Fazio R., Ekuakille A.L. (2017). A solar-powered white led-based uv-vis spectrophotometric system managed by pc for air pollution detection in faraway and unfriendly locations. Int. J. Smart Sens. Intell. Syst..

[17] Piasecki T., Breadmore M.C., Macka M. (2010). White LEDs as broad spectrum light sources for spectrophotometry: Demonstration in the visible spectrum range in a diode-array spectrophotometric detector. Electrophoresis.

[18] Kim J.S., Kim A.H., Oh H.B., Goh B.J., Lee E.S., Kim J.S., Jun J.H. (2015). Simple LED spectrophotometer for analysis of color information. Bio-Med. Mater. Eng..

[19] Gómez-Polo C., Gómez-Polo M., Celemin-Vinuela A., De Parga J.A.M.V. (2014). Differences between the human eye and the spectrophotometer in the shade matching of tooth colour. J. Dent..

[20] Francis F.J. (2017). Color measurement and interpretation. Instrumental Methods for Quality Assurance in Foods.

[21] Djouadi A., Kneur J.L., Moultaka G. (2007). SuSpect: A FORTRAN code for the supersymmetric and Higgs particle spectrum in the MSSM. Comput. Phys. Commun..

[22] Spectra FORTRAN Code. http://www.physics.sfasu.edu/astro/color/spectra.html.

[23] Stephenson D. (2016). A portable diode array spectrophotometer. Appl. Spectrosc..

[24] Jansen-van Vuuren R.D., Armin A., Pandey A.K., Burn P.L., Meredith P. (2016). Organic photodiodes: The future of full color detection and image sensing. Adv. Mater..

[25] Nie X., Ryckeboer E., Roelkens G., Baets R. (2017). CMOS-compatible broadband co-propagative stationary Fourier transform spectrometer integrated on a silicon nitride photonics platform. Opt. Express.

[26] Čekon M., Slávik R., Juras P. (2016). Obtainable method of measuring the solar radiant flux based on silicone photodiode element. Appl. Mech. Mater..

[27] Photodiode S12230–1, Sensitive to the Visible and Infrared Spectrum. http://cort.as/-Hgkl.

[28] Photodiode OSD15-E, Sensitive to the Visible Spectrum. http://cort.as/-Hgkq.

[29] Chun H., Rajbhandari S., Faulkner G., Tsonev D., Xie E., McKendry J.J.D., Haas H. (2016). LED based wavelength division multiplexed 10 Gb/s visible light communications. J. Lightwave Technol..

[30] LED VAOL-5EUV8T4 385 nm Wavelength Peak. http://cort.as/-Hbwt.

[31] Spectrophotometer V-5000 VIS. http://cort.as/-HglF.

[32] Min Y.K., Clauberg B., Hontelé B.J. (2003). LED Driver Circuit with PWM Output. U.S. Patent.

[33] Hu Y., Jovanovic M.M. (2008). LED driver with self-adaptive drive voltage. IEEE Trans. Power Electron..

[34] Dasgupta P.K., Eom I.Y., Morris K.J., Li J. (2003). Light emitting diode-based detectors: Absorbance, fluorescence and spectroelectrochemical measurements in a planar flow-through cell. Anal. Chim. Acta.

[35] Li L., Wang Z., Pei F., Wang X. (2013). Improved illumination for vision-based defect inspection of highly reflective metal surface. Chin. Opt. Lett..

[36] LED C503B-BCN-CW0Y04520–30 470 nm Wavelength Peak. http://cort.as/-Hbju.

[37] Komiyama R., Kageyama T., Miura M., Miyashita H., Lee S.S. Turbidity Monitoring of Lake Water by Transmittance Measuresment with a Simple Optical Setup. Proceedings of the 2015 IEEE SENSORS.

[38] LED SSL-LX5093UEGC 500 nm Wavelength Peak. http://cort.as/-HdZE.

[39] Lüders K., Pohl R.O. (2018). Diffraction. Pohl’s Introduction to Physics.

[40] Stone M. (2016). A Field Guide to Digital Color.

[41] Aydın C., Mansour S.A., Alahmed Z.A., Yakuphanoglu F. (2012). Structural and optical characterization of sol–gel derived boron doped Fe 2 O 3 nanostructured films. J Sol-Gel Sci. Technol..

[42] Coutsias E.A., Seok C., Dill K.A. (2004). Using quaternions to calculate RMSD. J. Comput. Chem..

